# Transition metal supported UiO-67 materials and their applications in catalysis

**DOI:** 10.3389/fchem.2025.1596868

**Published:** 2025-05-30

**Authors:** Tingting Li, Yan Li, Jingxin Mao

**Affiliations:** ^1^ Chongqing Key Laboratory of High Active Traditional Chinese Drug Delivery system, Chongqing Medical and Pharmaceutical College, Chongqing, China; ^2^ College of Pharmaceutical Sciences, Southwest University, Chongqing, China

**Keywords:** UiO-67, transition metal, catalyst, green chemistry, MOFs

## Abstract

Metal-organic frameworks (MOFs) have emerged as promising platforms for heterogeneous catalysis due to their tunable structures and high specific surface areas. Results indicate that modified composite MOFs not only exhibit superior water stability but also demonstrate broader applicability in catalysis, such as Fenton-like oxidation, Morita-Baylis-Hillman reactions, ethylene dimerization, and various photoelectrochemical processes. Among them, UiO-67, a zirconium-based MOF, has attracted extensive attention for its exceptional chemical stability, high catalytic activity, and well-defined microporous structure. This review introduces composites formed by different types of single and multi-metal loadings on UiO-67 and their demonstrated catalytic performance. It emphasizes the structure-performance relationships of these composites, highlighting how metal loading and spatial distribution influence their reactivity and stability. The current application status and existing challenges of UiO-67 series materials and their derivatives in catalysis are systematically reviewed. By integrating experimental results and mechanistic insights, this work underscores the transformative potential of UiO-67 series materials in meeting the demands of sustainable catalysis.

## 1 Introduction

In the 1990s, the concept of MOFs was introduced by Yaghi et al. (1995). At that time, MOFs had certain limitations in terms of pore size and stability. However, with the passage of time, MOFs, as an emerging material, have undergone remarkable transformations. Over time, MOFs, as an emerging material, have exhibited impressive transformations. Following optimization, MOFs have been capable of maintaining the integrity of their backbone even after the removal of guest molecules from their pores (Li et al., 1999). Furthermore, they have successfully undergone the transition from microporous to mesoporous structures (Rosi et al., 2003). Currently, MOFs have evolved into multifunctional materials characterized by high specific surface area, porosity, large pore size, stable physicochemical properties, and multi-metallic sites. These attributes have facilitated a diverse array of applications for MOFs in areas such as gas adsorption, catalysis, ion separation, and controlled drug release (Mukoyoshi and Kitagawa, 2022; Singh et al., 2021). Based on their structural characteristics, MOFs can be broadly classified into three categories: those containing nitrogen-heterocycles, those containing carboxyl groups, and those incorporating both nitrogen-heterocycles and carboxyl groups.

In recent years, MOFs have gained increasing favor among researchers. Notably, the MIL series, ZIF series, and UiO series of MOFs have received widespread attention. Among them, UiO series MOFs have demonstrated remarkable stability under high temperatures, high pressures, and in various solvent environments. It is noteworthy that although most MOFs exhibit unstable qualities in water, UiO series materials are surprisingly stable (Dong et al., 2020; Ogiwara et al., 2019; Piscopo et al., 2019). Therefore, researchers at home and abroad have used UiO series materials as a carrier to explore their catalytic applications by introducing metal ions or other structures to their structures for post-modification studies.

## 2 Synthesis and preparation of UiO-67

**FIGURE 1:**
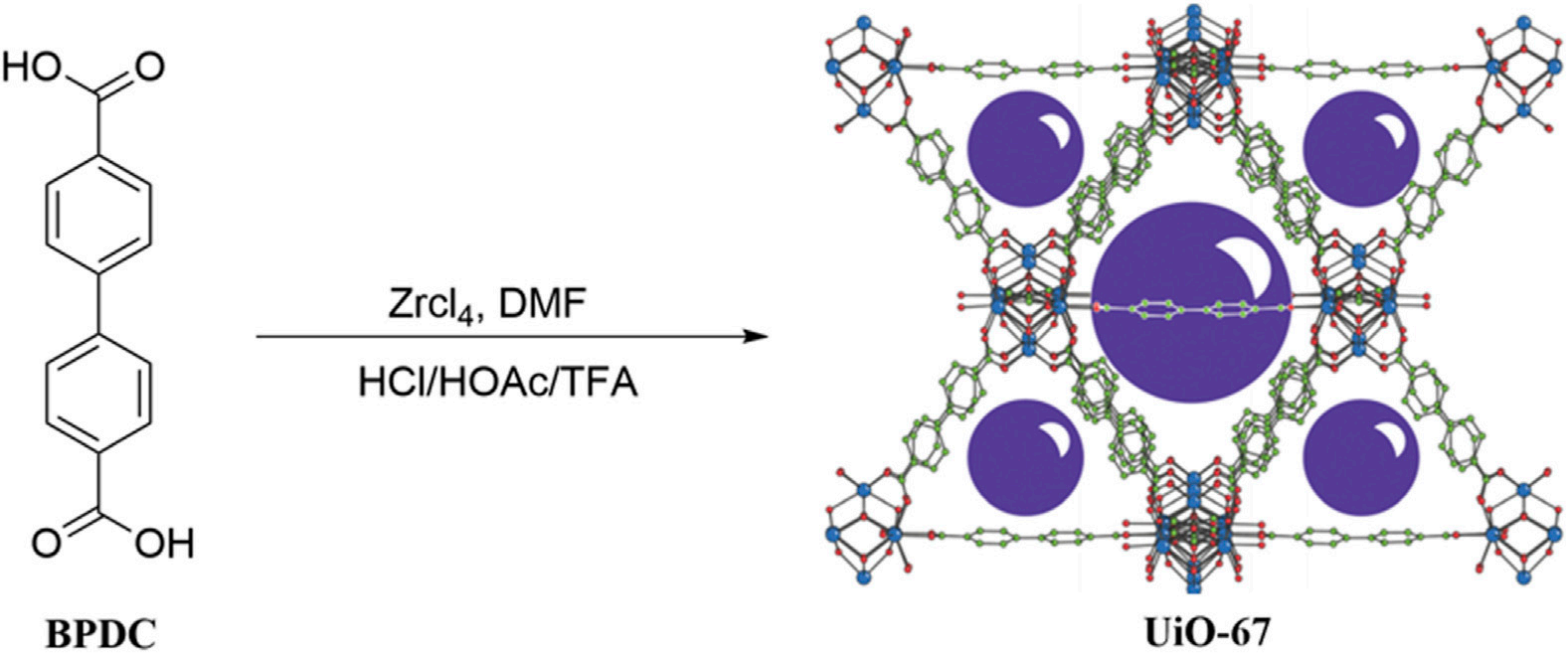
Synthesis pathway of UiO-67.

UiO-67, a white microcrystalline powder with the chemical formula Zr_6_O_4_(OH)_4_(BPDC)_6_, is typically synthesized through a hydrothermal method. The standard synthesis procedure is outlined below: Firstly, combine the ligand (4,4′-biphenyldicarboxylic acid), the metal compound (ZrCl_4_), and a conditioning acid (such as hydrochloric acid, acetic acid, trifluoroacetic acid, among others). Dissolve the mixture in DMF (N,N-dimethylformamide), then transfer it to a Teflon-lined stainless steel autoclave. Subject the mixture to a reaction for 72 h in a temperature-controlled oven set at 120°C. Upon completion of the reaction, the obtained product undergoes further processing. Subsequently, the product is filtered, washed, and dried to yield the final UiO-67 product (Figure 1).

It was revealed that UiO-67 exhibits a unique structural attribute, featuring octahedral cages that encompass eight tetrahedral cages, with octahedral holes dispersed across both its surface and edges (DeCoste et al., 2013). This peculiar configuration grants UiO-67 an exceptionally high surface area, which can reach as much as 2,200 m^2^/g (Cavka et al., 2008). Despite variations among researchers in synthetic details, including synthesis temperature, reaction duration, conditioning acid, and the selection of solvents during post-processing (Gutterød et al., 2019; Liu et al., 2018; Cliffe et al., 2017; Zhao et al., 2017), these differences did not have a significant impact on the fundamental structure of UiO-67. During the synthesis process, the addition of acid can effectively regulate the size and morphology of UiO-67, as well as control its nucleation rate, thus improving the reproducibility of the synthesis (Schaate et al., 2011). In contrast, if no acid is added during the synthesis, the specific surface area of UiO-67 is significantly reduced. In addition, in order to obtain a pure UiO-67 product, washing with DMF and low boiling solvents (e.g., methanol, acetonitrile, etc*.*) was required to remove unreacted ligands or benzoic acid and to displace the solvent DMF by low-boiling solvents. Lastly, the temperature and duration of the drying, which have a relatively small effect on the structure of the material, were aimed at removing solvents in the pore space for the purpose of activation.

## 3 Catalytic studies focusing on modifications of UiO-67

### 3.1 Loading of monometallic complexes on UiO-67

The utilization of MOFs as heterogeneous catalysts is gaining widespread attention, with an increasing focus on incorporating various metals into their frameworks. The literature documents several instances of successfully incorporating a single transition metal into UiO-67, where the modified ligands coordinate with metal ions to yield functionalized MOFs. In this manner, the potential applications of modified UiO-67 in areas like catalysis have been further explored and expanded (Sawano et al., 2015). Nevertheless, the field continues to confront numerous challenges, including the precise control of metal nanocrystal size, position, and arrangement order (Hou C. C. et al., 2015).

#### 3.1.1 Metallic Au and other metal complexes

Haruta et al. (1987) discovered that dispersed gold nanoparticles (Au NPs) exhibit catalytic activity for CO oxidation reactions at low temperatures, sparking widespread interest among researchers in Au nanocatalysts in 1987. With the accelerating advancement of MOFs, there has been speculation about whether loading Au nanoparticles onto MOFs also imparts catalytic activity. To validate this hypothesis, researchers have repeatedly explored this avenue. In recent years, a more refined approach for Au metallization and modification of UiO-67 materials has emerged, enabling their successful application in catalyzing a diverse range of chemical reactions, including redox reactions and cyclopropanation reactions, among others.

**FIGURE 2:**
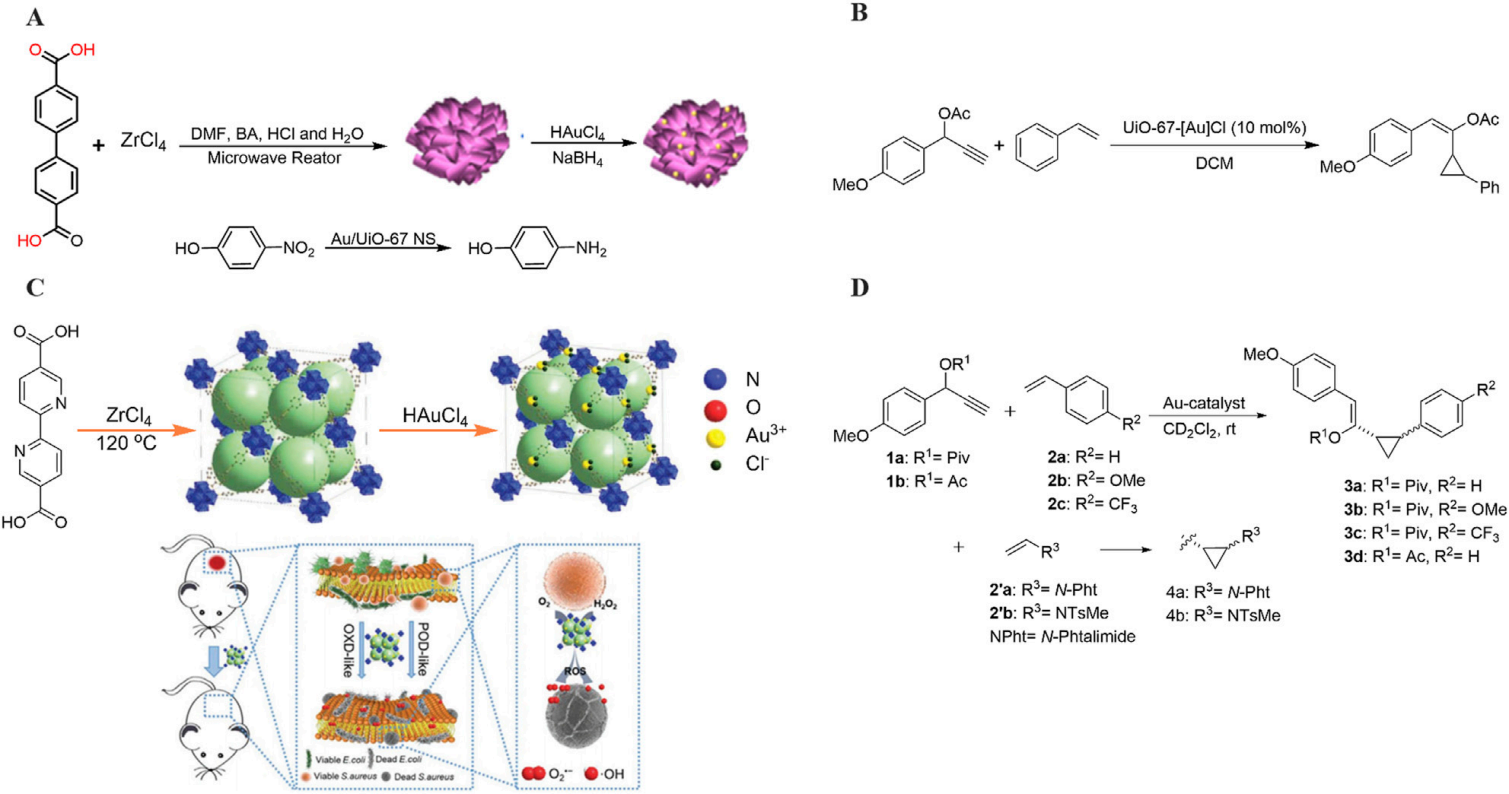
**(A)** Synthesis and catalytic reaction of Au/UiO-67 NS; **(B)** Synthesis and catalytic reaction of Au^3+^-N MOFs; **(C)** MOF-catalyzed reaction; **(D)** Studies on stereoselective cyclopropanation.

Liu et al. (2022) utilized benzoic acid and hydrochloric acid as modulating acids to swiftly synthesize two-dimensional Zr-MOFs, termed UiO-67 NS, through the application of microwave radiation in 2022 (Figure 2A). In comparison to the conventional solvothermal method, microwave radiation offers the advantages of rapidly and uniformly heating the reaction solution, thereby accelerating the chemical reaction process, markedly enhancing synthesis efficiency, and reducing the overall time required (Mbuya et al., 2022).

Subsequently, the researchers successfully synthesized Au/UiO-67 NS composites through *in situ* reduction of HAuCl_4_ within the pores of UiO-67 NS. The experimental findings demonstrated that the resulting nanocomposites exhibited remarkable catalytic activity for the reduction of 4-nitrophenol to 4-aminophenol. With a significant amount of research dedicated to developing synthetic nanomaterials that mimic the advantages of natural enzymes’ nanozymes, such as high catalytic activity, biocompatibility, and ease of modification and functionalization (Huang et al., 2019; Jiang et al., 2019; Wu et al., 2019), MOF-based nanozymes have emerged as a key area of focus in this field. In the same year, Pan et al. (2022) successfully coordinated Au^3+^ ions with bipyridine to synthesize Au^3+^-N MOFs materials possessing catalytic properties. It was discovered that these Au^3+^-N MOFs were capable of catalyzing the aerobic reduction of O_2_ to produce superoxide radical anions (O_2_^−^). Furthermore, they demonstrated peroxidase (POD) activity, forming hydroxyl radicals (⋅OH) in the presence of H_2_O_2_. The Au^3+^-modified UiO-67 excelled in catalyzing the production of reactive oxygen species (ROS), thereby enhancing antimicrobial activity without the need for supplementary oxidants. Notably, this material could also be utilized for wound healing, enabling aerobic conditions conducive to green synthesis (Figure 2B).

MOFs can be functionalized not only through the metallization of UiO-67 but also by incorporating pre-modified ligands. Levchenko et al. (2020) chose to anchor the synthesized ligand, Au(L) (OACF)_2_ [L = phenylpyridine dicarboxylic di-ester (PPYDE) or phenylpyridine dicarboxylic acid (PPYDC)], onto the UiO-67 backbone, resulting in a UiO-67-[Au]Cl composite. This composite exhibited exceptional catalytic performance in the cyclopropanation reaction (Figure 2C).

It was demonstrated that nearly all the complexes and MOFs exhibited catalytic activity towards the cyclopropanation products, achieving a conversion rate of 97% and demonstrating a preference for the generation of trans diastereoisomers. In contrast, the published literature typically reports that cis-trans isomerization proceeds at varying rates, ultimately leading to the formation of the cis-isomer of the cyclopropanation product (Figure 2D). It is conjectured that this preference for the cis-isomer in the literature may be closely linked to the specific metal complexes and secondary structures employed, as noted in reference (Reiersølmoen et al., 2018). In contrast, this MOF demonstrated high selectivity for the trans isomer in all cyclopropanation reactions, with the cis-to-trans structure ratio of the product remaining relatively constant throughout the reaction period. These findings indicate that UiO-67-[Au]Cl exhibits a unique stereoselectivity distinct from other Au(III) complexes and maintains its activity for cis-trans isomerization reactions throughout the entire course of the reaction.

#### 3.1.2 Metal Cu and other complexes

**FIGURE 3:**
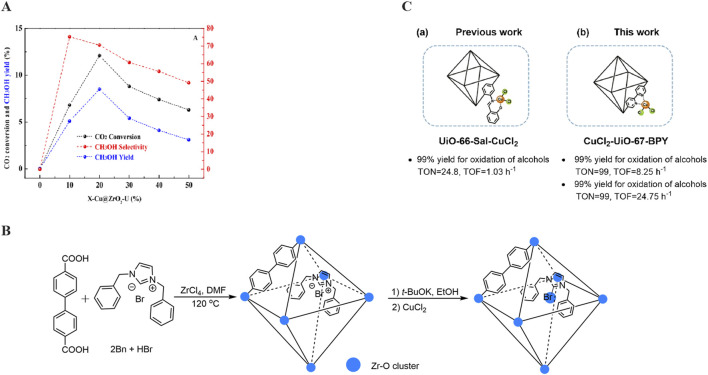
**(A)** Different copper loading capacities Cu@ZrO_2_-U Catalytic activity of catalysts for CO_2_ hydrogenation; **(B)** The Synthesis process of 2B_n_-Cu@UiO-67; **(C)** UiO-66-Sal-CuCl_2_ and CuCl_2_-UiO-67-BPY in aerobic oxidation reaction.

With the growing popularity of the concepts of “methanol economy” and “liquid sunshine,” the synthesis of methanol from hydrogen and carbon dioxide derived from renewable energy sources has emerged as one of the most promising approaches for carbon dioxide recycling (Zhong et al., 2020; Niu et al., 2022). Chen *et al.* (Zhong et al., 2018) utilized the deposition-precipitation (DP) method to prepare Cu@UiO-67, from which a series of Cu@ZrO_2_-U catalysts were subsequently derived in 2018. The experimental results revealed that the catalyst activity exhibited a volcano-like trend as the copper doping level increased (Figure 3A). Notably, 20-Cu@ZrO_2_-U demonstrated optimal performance in terms of CO_2_ conversion, methanol selectivity, and yield, suggesting that a copper content of 20 wt% is the most suitable for the preparation of this catalyst. Furthermore, the abundant presence of Cu^+^ and lattice oxygen in the catalyst promotes the formation of the Cu^+^-ZrO_2_ interface, which is a crucial factor in the synthesis of methanol through CO_2_ hydrogenation.

Similarly, Chen et al. (2022) ligated copper single-atom sites (Cu SAS) with N-heterocyclic carbene (NHC) embedded within UiO-67 to obtain the catalyst 2Bn-Cu@UiO-67 (Figure 3B). This catalyst can be employed in electrochemical reactions for the reduction of carbon dioxide (CO_2_) to methane. In this material, the enrichment of N-heterocyclic carbene molecules (NHCs) further enhances the surface charge density of the heterogeneous metal single-atom sites (SASs), thereby intensifying the electrophilic binding and conversion of CO_2_. This catalyst design strategy not only offers a novel approach for designing electrocatalytic carbon dioxide reduction reactions but may also provide valuable insights for research in other related fields.

Meanwhile, Cu-loaded UiO-67 can also catalyze aerobic reactions, such as the selective oxidation of alcohols and the epoxidation of olefins. Li R. et al. (2022) successfully synthesized Cu@UiO-67-BPY metal-organic framework materials using a one-pot method in 2022. In a prior study (Hou J. et al., 2015), the research group conducted post-synthetic modification of zirconium-based MOFs UiO-66-NH_2_ using salicylaldehyde and immobilized CuCl_2_ onto the surface of the functionalized UiO-66-NH_2_, yielding the UiO-66-Sal-CuCl_2_ material. This catalytic material exhibited effective catalysis in the selective oxidation of benzyl alcohol, achieving a turnover number (TON) of 24.75 and a turnover frequency (TOF) of 1.03 h^−1^ (Figure 3C). Unfortunately, its application has not yet been expanded to epoxidation reactions. By analyzing the characterization results of the CuCl_2_-UiO-67-BPY material in relation to the oxidation of benzyl alcohols catalyzed by various materials, it can be deduced that the presence of the halogen anion facilitates the oxidation process, as the chlorine radical, with its strong electronegativity, aids in the removal of hydrogen. Furthermore, the CuCl_2_-UiO-67-BPY catalyst can be recovered and reused at least ten times without significantly compromising its yield or selectivity.

#### 3.1.3 Metal Pd and other complexes

Functionalizing noble metals with MOFs represents a highly promising synthetic approach to combining the porosity and high specific surface area of MOFs with the elevated catalytic activity of noble metals. This results in the synthesis of catalytic materials that exhibit high catalytic activity while simultaneously reducing catalytic costs (Bugaev et al., 2019). Palladium (Pd), a noble metal, is widely utilized in the preparation of palladium catalysts, which are commonly employed in the chemical industry. These catalysts offer the advantages of high catalytic activity, high selectivity, and low usage, but they also suffer from the disadvantage of a high price. Consequently, numerous researchers have coordinated palladium compounds with MOFs to achieve high catalytic activity while incorporating the benefits of cost reduction.

**FIGURE 4:**
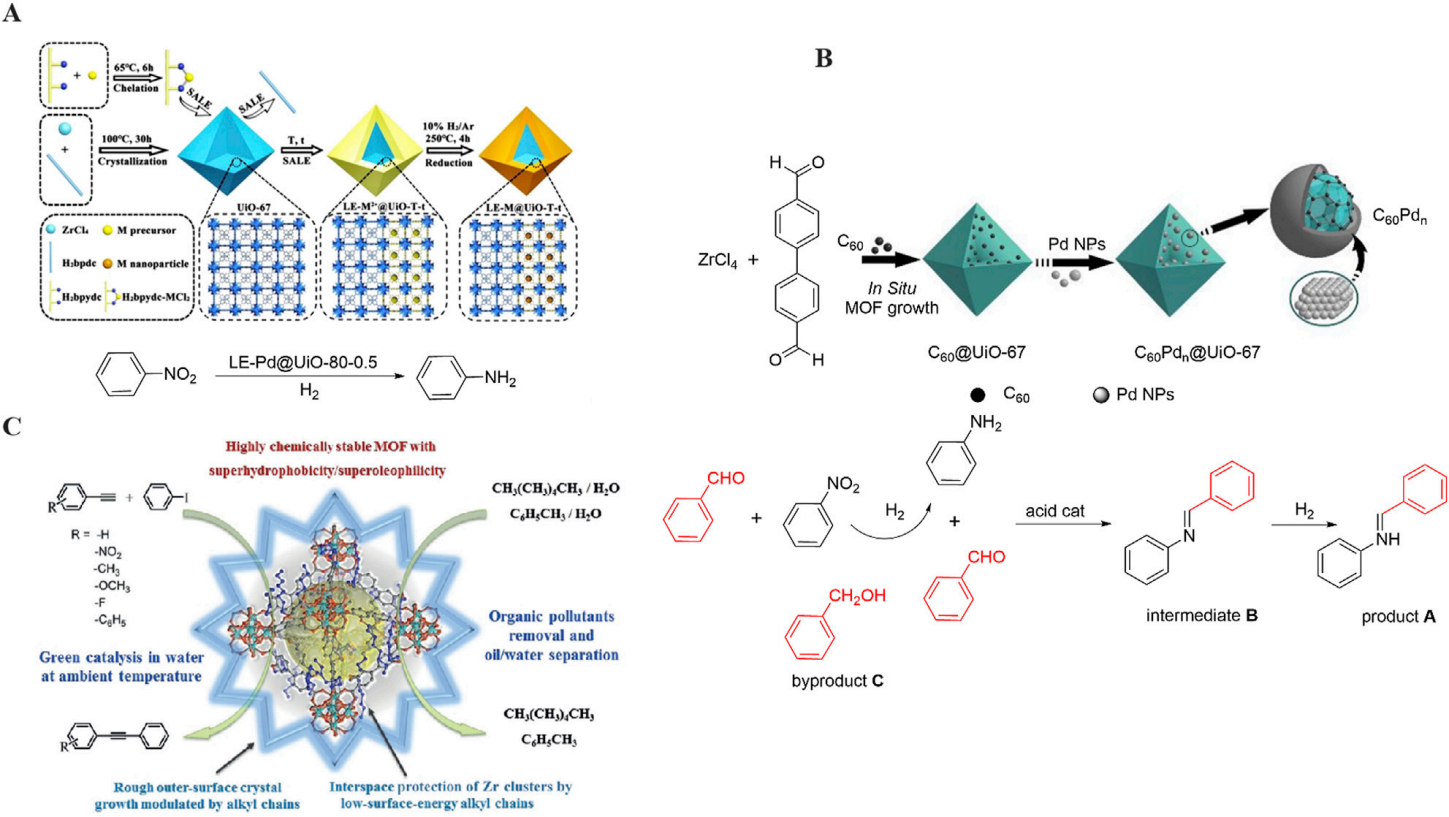
**(A)** Synthesis and catalytic reaction of LE-Pd@UiO-80-0.5; **(B)** Synthesis and catalytic reaction of C_60_Pd_n_@UiO-67; **(C)** Catalytic reaction of UiO-67-Oct-L_2_-X%-Pd_II_.

Cui et al. (2021) introduced a solvent-assisted ligand exchange-hydrogen reduction (SALE-HR) strategy to selectively encapsulate ultrafine palladium nanoparticles within the shallow structure of a MOF, specifically UiO-67 in 2021. By precisely controlling factors such as temperature and reaction duration, the thickness of the embedded layer and the size of the metal nanoparticles can be accurately regulated (Figure 4A).

The experimental results demonstrate that the LE-Pd@UiO-80-0.5 composite materials, featuring the thinnest Pd embedding layer, exhibit superior catalytic performance in the hydrogenation reaction of nitroaromatic hydrocarbons. Specifically, nitrobenzene can be nearly fully converted to aniline, achieving a conversion frequency as high as 600 h^-1^. This may be attributed to the fact that the metal nanoparticles are embedded as closely as possible to the outer surface of the MOFs, thereby reducing the diffusion distance and consequently enhancing the catalytic activity and utilization efficiency of the LE-Pd@UiO-80-0.5 material.

Similarly, Zheng D. Y. et al. (2018) successfully modified UiO-67 through Pd metallization, which was subsequently employed to catalyze the hydrogenation reaction. Initially, C_60_ molecules were encapsulated within the UiO-67 framework, after which Pd NPs were introduced onto C_60_@UiO-67 utilizing a direct immersion-stirring synthesis method. The method is straightforward, efficient, and significantly enhances the hydrogenation activity of the catalytic reaction owing to the synergistic effect between UiO-67, the encapsulated carbon C_60_, and the Lewis acid sites provided by UiO-67 (Figure 4B). In comparison to previous literature, C_60_Pd_n_@UiO-67 achieves the same conversion with a shorter duration and milder reaction conditions. The preparation method of this composite paves a new avenue for designing high-catalytic-activity composites based on MOFs, potentially inspiring novel ideas for the construction of functional materials that integrate MOFs with fullerene materials. In addition, Zhu et al. (2019) synthesized (super) hydrophobic MOFs, UiO-67-Oct-L_2_-X%-PdII, by incorporating alkyl chains and Pd(II) into the UiO-67 framework. This approach serves a dual purpose: it safeguards the hydrophilic Zr_6_O_8_ clusters, allowing the MOFs to modulate the surface roughness of the crystal morphology, and endows them with superoleophilicity, thereby achieving superhydrophobicity. Notably, these MOFs can catalyze the Sonogashira reaction at room temperature, exhibiting excellent catalytic efficiency and recyclability (Figure 4C).

#### 3.1.4 Metal Pt and other complexes

In the realm of addressing carbon dioxide pollution, catalytic hydrogenation reactions and methane reactions constitute the primary research directions (Li et al., 2018; Kattel et al., 2017; Chen X. et al., 2017). Within these reactions, the interplay between metal nanoparticles and MOFs materials holds immense significance for enhancing the catalytic activity and selectivity of the processes.

**FIGURE 5:**
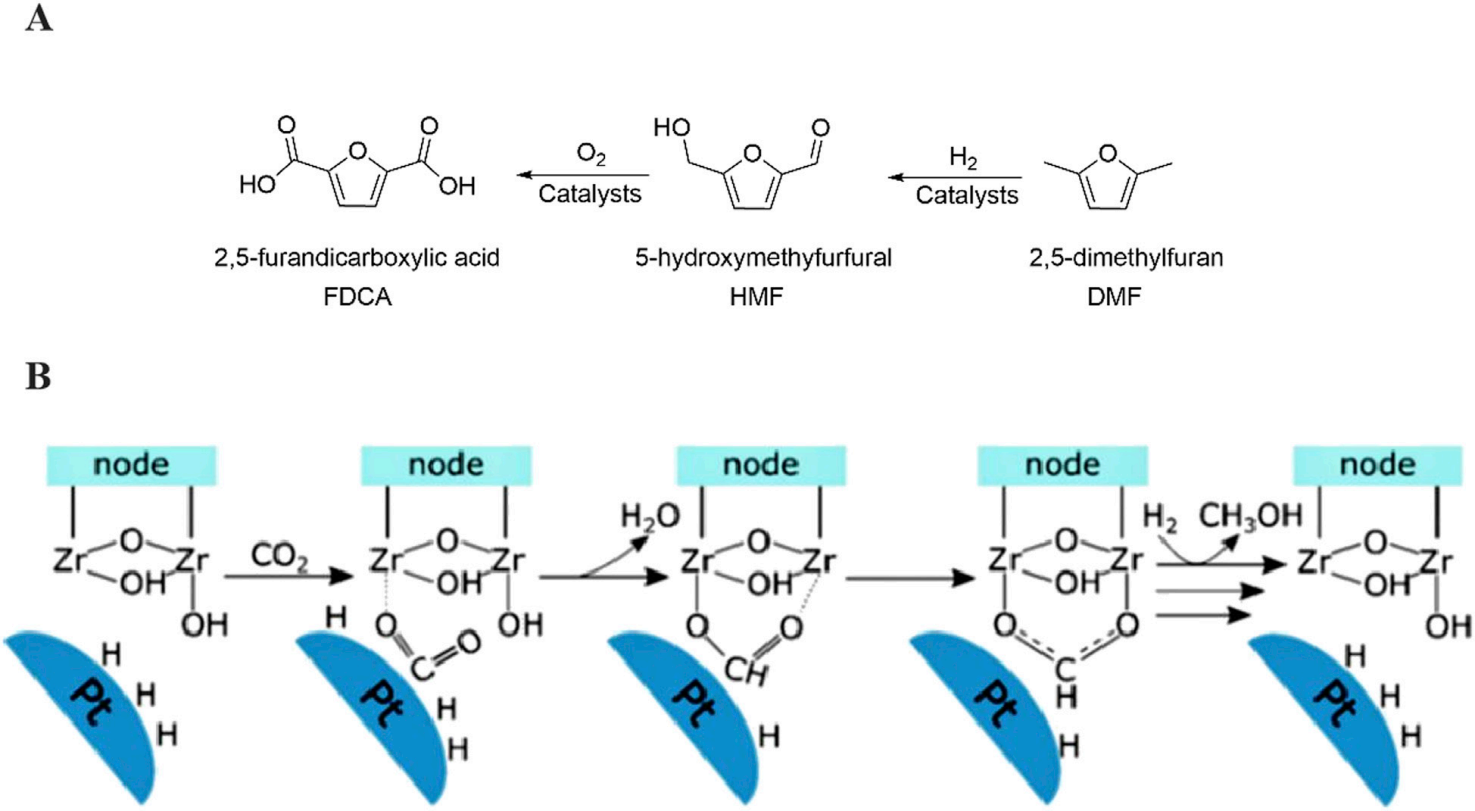
**(A)** Catalytic hydrogenation reaction; **(B)** Schematic diagram of the mechanism of carbon dioxide hydrogenation reaction in methanol formation at the Pt-Zr node interface.

Hester et al. (2016) successfully loaded Pt nanoparticles (NPs, 0.5 wt%) onto zirconium-based metal-organic frameworks, resulting in the Pt catalyst NP@UiO-67 (dark gray) in 2016. Based on previous literature, this study marks the first in-depth examination of the stability and redox performance of catalysts modified using UiO-series MOFs during the catalytic process. According to previous literature, this represents the first comprehensive investigation into the stability and redox performance of catalysts modified with UiO-series MOFs during the catalytic process. To assess the thermal performance of this composite material, thermogravimetric analysis (TGA) combined with differential thermal analysis (DTA) was employed to compare the UiO-67 composite material with pure UiO-67. The experimental findings revealed that the incorporation of Pt led to a decrease in the thermal stability of the material. However, it is gratifying to note that Pt NP@UiO-67 exhibited higher activity and selectivity in the hydrogenation reaction (Figure 5A). The likely explanation for this phenomenon is that the incorporation of Pt nanoparticles significantly enhances the chemical adsorption of H_2_ on UiO-67 at 323 K.

Gutterød et al. (2020) investigated the loading of 3 wt% Pt nanoparticles onto zirconium-based metal-organic frameworks (UiO-67), resulting in UiO-67 Pt in 2020. By enhancing the number of defects in the Zr nodes, the production rates of methanol and methane could be substantially increased. Following this, the team employed infrared steady-state and transient dynamics, along with spectral and density functional theory (DFT) modeling studies, to thoroughly examine the mechanism of UiO-67-Pt-catalyzed carbon dioxide hydrogenation (Gutterød et al., 2019) (Figure 5B). Previous research has shown that methanol is formed at the interface between defective Zr nodes and Pt nanoparticles (NPs) through the intermediation of formate species attached to the Zr nodes. Besides the activation of hydrogen on Pt NPs, the mechanism of methanol formation is distinct from that of by-product formation, specifically carbon monoxide (CO) and methane.

As a typical precious metal catalyst, Pt has not only been utilized in catalyzing the hydrogenation of carbon dioxide but has also garnered attention in the commercial production of silicon products through silicification reactions (Tondreau et al., 2012). In 2022, Wei et al. (2022) employed a dual ligand-assisted [2,2′-Bipyridine,5,5′-dimethyl (H_2_bpydc) and 4,4′-Biphenyldicarboxylic acid (H_2_bpdc)] strategy to synthesize a Pt/UiO-67-bpdc catalyst for catalyzing the reaction between alcohols and silane (Table 1). Due to the robust coordination between Pt^2+^ and pyridine on the UiO-67 backbone, highly dispersed Pt sites can be achieved. Additionally, the incorporation of the inert 4,4′-biphenyldicarboxylic acid ligand enhances the overall stability of the material. Conversely, in the absence of this ligand, the Pt/UiO-67-bpdc precursor is susceptible to aggregation during pyrolysis, leading to the formation of Pt nanoparticles. The experimental results demonstrated that the catalyst possessed a high Pt loading content of 0.6962 wt%. During the silane oxidation process, when the Pt loading was decreased to 0.005%, the Pt SAC/N-C catalyst, obtained through high-temperature pyrolysis and acid leaching treatment, exhibited a remarkable transition frequency (TOF) of 9,920 h^−1^. This catalyst was successfully employed for the efficient formation of silica-oxygen bonds.

**TABLE 1:** Pt SAC/N-C catalyzed reaction between alcohol and silane.

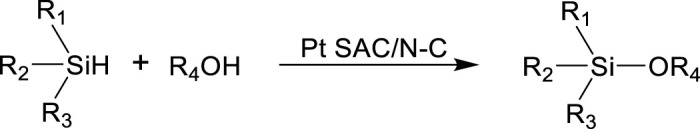				
Entry	Alcohols	Silanes	Reaction time (h)	Conversion (%)^b^
1	Ethanol	Me_2_PhSiH	3	99.0
2	1-Butanol	Me_2_PhSiH	3	98.2
3	Ethanol	Et_3_SiH	3	99.8
4	1-Butanol	Et_3_SiH	3	98.6
5	Ethanol	Me_2_ (*t*-Bu)SiH	3	99.5
6	1-Butanol	Me_2_ (*t*-Bu)SiH	3	98.8
7	Ethanol	Ph_2_SiH_2_	3	97.5
8	1-Butanol	Ph_2_SiH_2_	3	96.4

^*a*^ Reaction conditions: Catalyst Pt SAC/N-C (0.005 mmol of Pt, based on ICP, analysis), silane (10.0 mmol), and alcohols (3.0 mL) were added sequentially to a 10.0 mL round-bottom flask. The mixture was then stirred at 40 °C under a nitrogen atmosphere for 3 h.

^*b*^ Conversion was determined by GC, analysis.

#### 3.1.5 Other metal complexes

The UiO-67 series of MOFs, due to their exceptional thermal stability, are ideal candidates for exploring synthetic functionalization pathways that aim to achieve high catalytic activity while minimizing catalyst costs. It is worth noting that, although noble metals are less commonly utilized as metal nodes in the preparation of MOFs, functional organic ligands possess a significant advantage in coordinating metal ions. Their robust anchoring effect can effectively stabilize noble metal monoatoms. Therefore, numerous researchers have dedicated their efforts to incorporating noble metals (such as Pd, Au, Pt, and Ru) into the UiO-67 metal-organic framework. Furthermore, previous studies have successfully incorporated various metal ions and functional organic ligands (e.g., Al, Ce, Cu, Fe, Ir, Mo, Ni, etc*.*) into UiO-67 frameworks. Subsequent post-synthetic modification techniques have further expanded the catalytic applications of UiO-67. A summary of representative UiO-67-based MOFs for catalytic purposes is provided in Table 2.

**TABLE 2:** Application of UiO-67 series MOFs materials in the field of catalysis.

Materials	Synthesis method	Catalytic applications	Literatures
UiO-66@UiO-67-BPY-Ag	Solvothermal synthesis	Knoevenagel	Gong et al. (2019)
Al@UiO-67	Solvothermal synthesis	Meerwein-Ponndorf-Verley	Larson et al. (2018)
UiO-67-Ce	Solvothermal synthesis	photocatalytic hydrogenation	An et al. (2019)
Ce-doped UiO-67-400	Hydrothermal synthesis	Fenton-like oxidation	Dong et al. (2021)
Co-UiO-67、Re-UiO-67	Solvothermal synthesis	Photocatalytic reduction of carbon dioxide	Gao et al. (2020)
Ni-UiO-67-bpy_11%_	Solvothermal synthesis	Ethylene dimerization	Kømurcu et al. (2021)
Ni-doped UIO-67	Hydrothermal synthesis	Hydrogen Evolution Reaction	Shah et al. (2021)
Ni@UiO-67-NN-P	Solvothermal synthesis	Degradation of methyl orange dye	Li et al. (2020)
UiO-67@Fe	Solvothermal synthesis	Morita-Baylis-Hillman	Zhao et al. (2022a)
FeCl_3_/UiO-67bpy	Solvothermal synthesis	Catalytic hydrolysis of 5-hydroxymethoxyfurfural	Zhang et al. (2021)
UiO-67-Mix-Ir	Solvothermal synthesis	Boronylation	Yang B. et al. (2019)
UiO-67-supported Ir(C_2_H_4_)_2_	Solvothermal synthesis	Ethylene dimerization	Yang et al. (2020)
UiO-67-MoO(O_2_)_2_	Solvothermal synthesis	Oxidation of cyclohexane	Hong et al. (2020)
RuB-RuTB-UiO-67	Solvothermal synthesis	Photoelectrochemical oxidation of benzyl alcohol	Lin et al. (2021)
UiO-67-[RuOH_2_] @FTO	Solvothermal synthesis	Electrocatalytic water oxidation reaction	Johnson et al. (2017)
Ru-UiO-67	Solvothermal synthesis	Catalytic water oxidation	Lin et al. (2018)
Rudcbpy-UiO-67(Zr)	Solvothermal synthesis	Photocatalytic debromination reduction	Santiago-Portillo et al. (2018)
TiO_2_@UIO-67-Zr/Ti	Solvothermal synthesis , Microwave	Photocatalytic oxidation of 5-hydroxymethoxyfurfural	Zhou et al. (2021)
Rh(C_2_H_4_)_2_ on UiO-67	Solvothermal synthesis	Ethylene hydrogenation and dimerization	Bernales et al. (2017)
Bpy-UiO-Ir	Solvothermal synthesis	C-H bond boronation of aromatic hydrocarbons	Manna et al. (2014)

### 3.2 Loading of polymetallic complexes on UiO-67

Numerous studies have confirmed that multimetallic nanoparticles often display electronic and chemical properties that are distinctly different from those of monometallic nanoparticles. By combining multiple metals, a synergistic effect can be achieved, thereby enhancing the stability and catalytic activity of the materials (Ferrando et al., 2008; Chen et al., 2005). For instance, bimetallic nanoparticles (BNPs) are renowned for their exceptional catalytic activity in CO_2_ hydrogenation.

**FIGURE 6:**
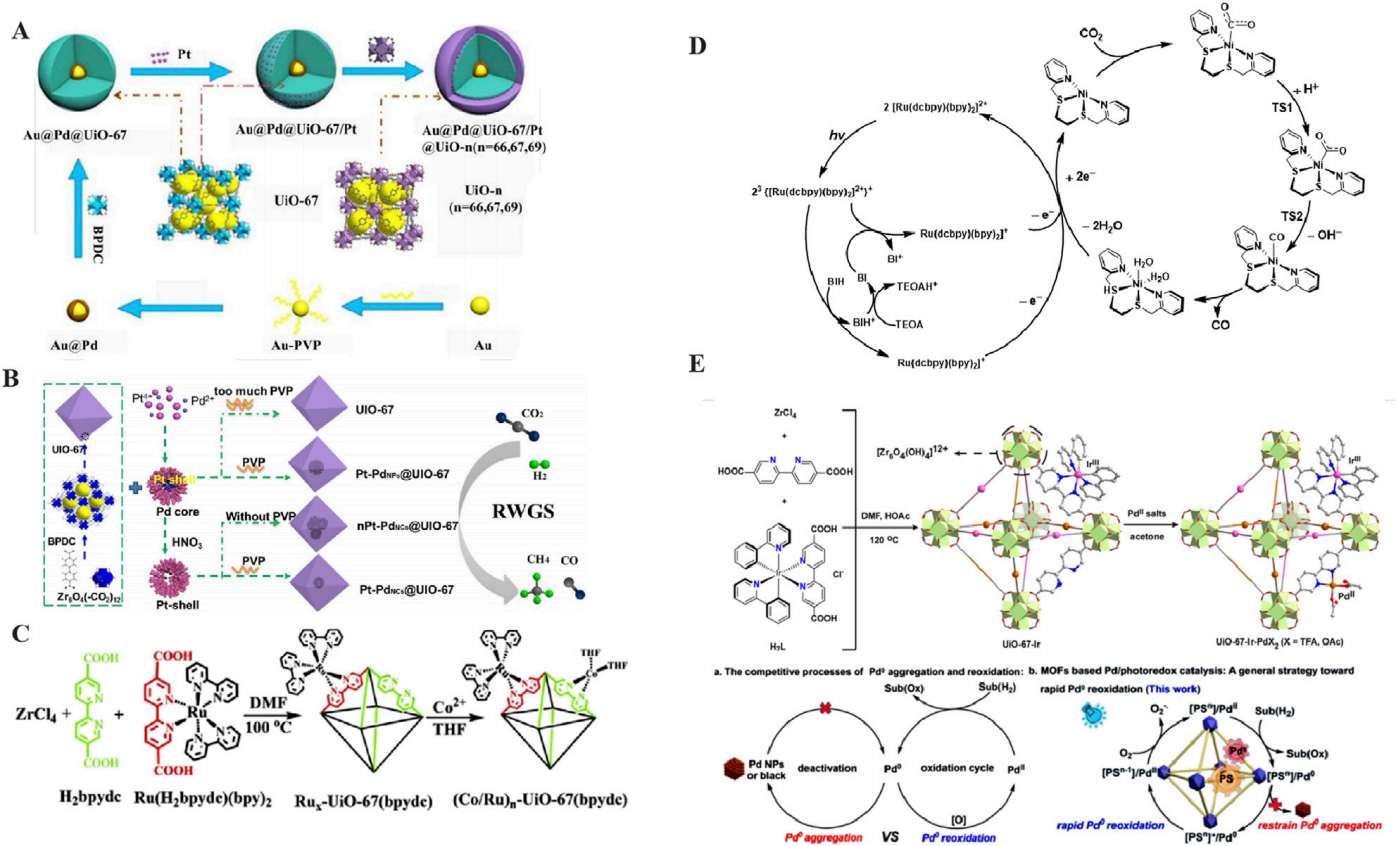
**(A)** The synthesis route of Au@Pd@UIO-67/Pt@UIO-n NPs; **(B)** (Co./Ru) N-UiO-67 synthesis process schematic diagram; **(C)** Synthesis and catalytic reaction of MOF; **(D)** The Mechanism of photocatalytic reduction of carbon dioxide to CO in Ni@Ru-UiO-67/BIH/TEOA system; **(E)** Synthesis and catalytic reaction of MOF.

Xu et al. (2019) successfully synthesized a spherical sandwich catalyst in 2019 (Figure 6A). The material featured Au NPs at its core, with Au@Pd NPs encapsulated within the center of the spherical structure. Subsequently, Pt nanoparticles were loaded onto the surface of this material, resulting in an Au@Pd@UIO-67/Pt composite. Finally, after undergoing additional coating treatment, the ultimate catalyst, Au@Pd@UIO-67/Pt@UiO-67, was obtained. This catalyst was reported to substantially enhance the catalytic activity for CO_2_ conversion. Furthermore, the palladium layer adhered to the gold core served to prevent the oxidation of the gold, thereby further improving the stability and activity of the catalyst (Zheng Z. et al., 2018).

In the same year, Liu et al. (2019) also succeeded in synthesizing MOFs loaded with polymetallic nanoparticles, enabling photocatalytic carbon dioxide reduction to syngas (CO and H_2_). The team proposed a straightforward two-step self-assembly process to successfully load Co and Ru onto UiO-67, yielding the catalyst (Co/Ru)_n_-UiO-67(bpydc) (Figure 6B). By meticulously adjusting the water content and the Co/Ru ratio, they were able to effectively control the H_2_ to CO ratio. When the Co/Ru ratio is set to 2.4 and the water content is maintained at 10%, the reaction catalyzed by (Co/Ru)_2.4_-UiO-67(bpydc) yields a syngas with an H_2_:CO ratio of 2:1. Under these optimal conditions, the yield of high-efficiency syngas can reach as high as 13,600 μmol g^-1^ in 16 h, surpassing the yields achieved by comparable homogeneous catalytic systems. Furthermore, Zhang H. et al. (2020) prepared the octahedral material M@UiO-67 (M = Pt-Pd NPs, Pt NPs), which demonstrated exceptional performance in terms of CO_2_ conversion and CO selectivity in RWGS in 2020. Experimental findings indicated that the introduction of acetic acid exerted a marked effect on the morphology of the M@UiO-67 framework. Moreover, polyvinylpyrrolidone (PVP) was shown to effectively regulate both the structural features and dimensional properties of the composite material. (Figure 6C).

Yan et al. (2019) synthesized the composite photocatalyst Ni@Ru-UiO-67, notable for its high activity and selectivity. This photocatalyst can reduce carbon dioxide to CO under visible light illumination, achieving a TON of 581 and a selectivity of up to 99%. Furthermore, the photocatalytic mechanism of Ni@Ru-UiO-67 was thoroughly investigated in this study (Figure 6D). By integrating the findings from ultrafast transient absorption spectra with theoretical calculations, the high catalytic activity observed is attributed to the efficient charge transfer process occurring between Ru-UiO-67 and Ni(II) complexes.

To address the issue of palladium reoxidation, Li J. et al. (2022) incorporated the photosensitizers Ir(III)PS and Pd(II) within the UiO-67 material, thereby constructing a novel molecular conformal material designated as UiO-67-Ir-PdX_2_ in 2022. Due to the stabilizing effect of the MOFs framework on the metal sites Pd and Ir, as well as the optimal distance between them which facilitates rapid electron transfer, UiO-67-Ir-PdX_2_ exhibits a frequency of Pd-catalyzed conversion under visible light that is 25 times higher compared to existing catalytic systems. The MOF was successfully utilized in a Pd-catalyzed oxidation reaction, fulfilling the objectives of minimizing Pd metal consumption and ensuring recyclability. During the oxidation process, the synergistic effect of Pd^0^ aggregation and the concurrent re-oxidation process facilitated an efficient catalytic cycle (Figure 6E). Specifically, the excitation of Pd^0^ catalysts can serve to decrease the activation energy required for the oxidative addition step. Furthermore, this study anticipates the extension of this strategy to a broader spectrum of transition metals, such as Ru and Rh, offering novel insights for future catalyst design endeavors.

## 4 Discussion

In recent years, the pharmaceutical industry has increasingly emphasized green chemistry, amidst growing global environmental concerns and gradual energy shortages. The objective is to design synthesis methods for compounds that maximize the incorporation of all materials from the production process into the final product (Sheldon, 2016; Tarasova et al., 2016). For instance, catalysts, which are frequently utilized in chemical reactions, are classified into homogeneous and heterogeneous catalysts based on the phase state of the reaction system (Zhu et al., 2022). The biggest advantage of non-homogeneous catalysts is that they are easy to be separated from the reaction system, which is relative to homogeneous catalysts, and the disadvantage is that the catalytic efficiency is mostly inferior to homogeneous catalysts, and the reaction is not as easy to be controlled as that of homogeneous catalysts (Poovan et al., 2022), whereas MOFs, as a kind of non-homogeneous catalysts, combine the advantages of non-homogeneous catalysts while compensating for their shortcomings. MOFs possess the following advantages: (1) Their synthesis is straightforward, as the reactivity of metal ions with carboxylic acids and nitrogen-containing heterocyclic ligands is exceptionally high. Additionally, the reaction conditions are mild, allowing for the synthesis of most MOFs in a single step via the solvothermal method. (2) The functional groups and coordination properties of the ligand can be flexibly altered due to the electrostatic interactions between Lewis acids and metal ions. (3) The metal ions that act as backbone vertices have two roles: on the one hand, they act as nodes to provide the backbone’s pivot, and on the other hand, they form branches at the pivot nodes, which allow the MOFs backbone to be extended, thus enhancing the physical properties (e.g., porous and chiral) of the MOFs and forming a multidimensional spatial structure (He et al., 2021). (4) When compared with traditional homogeneous catalysts, it can be easily separated and recycled from the reaction system, thereby enabling multiple and repeated cyclic catalytic processes. This characteristic holds significant importance in green catalytic synthesis.

Numerous studies have demonstrated that MOFs exhibit superior performance across diverse applications when compared to traditional porous solid materials, including zeolites and carbon-based porous materials, as referenced in study (Bai et al., 2016). The metal ions within the structural framework of UiO-67 form robust chemical bonds with the organic ligands, endowing it with the ability to withstand structural damage to a certain degree even at elevated temperatures. This attribute grants UiO-67 exceptional high thermal stability, allowing it to maintain its crystalline structure and porous characteristics largely intact when exposed to temperatures below 200°C. Concurrently, these materials possess a significantly larger specific surface area than molecular sieves with comparable pore structures, and they retain the integrity of their backbone even after the solvent molecules within the pores are removed, as indicated in studies (He et al., 2021; Valdebenito et al., 2022; Thür et al., 2019). Zirconium, being abundant in nature and present in all biological systems, along with its low toxicity, further enhances the prospects for the development and application of Zr-MOFs. UiO-66 and UiO-67 are both renowned MOFs. In comparison to UiO-66, UiO-67 structurally substitutes terephthalic acid (bdc) with biphenyl-4,4′-dicarboxylic acid (bpdc). Given that the biphenyl group is elongated relative to the benzene ring, this substitution results in a relatively larger pore size for UiO-67. The enlarged pore size facilitates the diffusion and adsorption of macromolecules, enabling its utilization in the separation or loading of larger molecular species. Consequently, UiO-67 also features a more uniform pore architecture, which empowers it to engage more selectively with specific magnetic guest molecules during adsorption and separation processes, thereby enhancing separation efficacy and adsorption specificity. The UiO-67 series is also celebrated for its surface tunability, stemming from its distinctive structure and chemical composition. These attributes enable the surface to be functionalized through a multitude of approaches, allowing for the customization of its affinity towards various substances, the creation of catalytic active sites, and more, all tailored to meet diverse application demands. In contrast, UiO-66 exhibits a slightly greater degree of versatility and flexibility when it comes to surface modification. Meanwhile, upon undergoing surface modification, UiO-67 exhibits a surface energy that is more favorable for the binding or dispersion of other substances in certain applications. Furthermore, it catalyzes a broader spectrum of reaction types, particularly in the realm of photoelectrocatalytic hydrogen production, as documented in studies (Bai et al., 2016; Liu, 2020).

Regrettably, we found through extensive literature research that the vast majority of the articles barely investigated the catalytic mechanism. The reason for this is: (1) The structure and composition of MOFs are intricate. Their active sites may be located at the metal center, on a specific group of the ligand, or result from a synergistic effect between the two. Furthermore, the significant variations in pore structure, pore size, and surface properties among different MOFs influence the adsorption of reactant molecules and the reaction pathways, complicating the clear definition and uniform description of the reaction mechanism. Additionally, some MOFs exhibit diverse metal-ligand coordination modes, with the substrate activation mode differing across these modes, further obscuring the determination of the dominant reaction mechanism. (2) Influence of multifaceted factors in the catalytic process: Within MOF catalytic reactions, not only does the intrinsic nature of the MOF itself play a role, but external reaction conditions—such as temperature, pressure, solvent type, and reactant concentration—also exert an influence on the reaction mechanism. Concurrently, MOFs may undergo structural transformations or protonation/deprotonation events during the reaction, thereby augmenting the complexity of mechanism elucidation. (3) Constraints of characterization methodologies: Elucidating the reaction mechanism necessitates the utilization of diverse characterization techniques to glean insights into the intermediary states and active sites involved in the reaction. Nevertheless, contemporary characterization methods are not without their limitations, which hinder the ability to monitor the MOF catalytic reaction process *in situ*, in real time, and with precision. (4) Challenges and substantial expenses associated with mechanism investigation: Delving into the intricacies of MOF catalytic reaction mechanisms demands an integration of experimental methodologies and theoretical computations, encompassing quantum chemical calculations, molecular dynamics simulations, among others, alongside a multitude of controlled experiments. This endeavor is both time-intensive and resource-demanding, incurring significant costs. Certain studies may prioritize practical applications, such as catalyst synthesis, catalytic efficacy, and selectivity, thereby neglecting an in-depth exploration of the reaction mechanism due to constraints imposed by research timelines and resource availability. Consequently, these mechanisms may remain unexplored and thus, are excluded from the article. (5) Ambiguity and discordance surrounding the mechanism: Despite the existence of pertinent research, the mechanism underlying MOF catalytic reactions may remain shrouded in ambiguity or rife with controversy. Varying experimental conditions and research methodologies employed by different research groups often culminate in divergent conclusions, prompting authors to eschew listing the reaction mechanism in favor of emphasizing more definitive research outcomes, such as catalytic performance. This strategic decision is made to circumvent potential controversies or in instances where the evidence base is insufficiently robust.

In this paper, we discuss the structural properties and catalytic activities of functionalized UiO-67 in general terms, and review the research progress in post-loading transition metal catalysis utilizing this material. Currently, UiO-67 and its structurally modified variants are primarily utilized in applications such as gas separation (Wang et al., 2014; Yan et al., 2021) and storage (Zhao et al., 2022b; Filippousi et al., 2016), drug delivery (Fang et al., 2022; Abazari et al., 2021), heterogeneous catalysis (Ren et al., 2021; Zhang S. et al., 2020; Zhang T. et al., 2020), electrochemistry (Dai et al., 2019; Zhang et al., 2018), and adsorption (Gong et al., 2019; Zhao et al., 2022c). In catalytic applications, UiO-67 materials are frequently employed as carriers for heterogeneous catalysts due to their exceptional stability, multiple coordination sites, ease of functionalization, and high surface area (Tahmouresilerd et al., 2018; Wei et al., 2018; Yang X. et al., 2019). There are four main pathways for the synthesis of UiO-67: Modulated synthesis (Cavka et al., 2008), Isoreticular expansion (Bae et al., 2010; Liu et al., 2015), Topology-guided design (Gutov et al., 2014), Postsynthetic functionalization (Savonnet et al., 2010). Typically, the catalytically active component can be incorporated into the MOFs framework through the method of ligand pre-modification, where the pre-modified ligand subsequently coordinates with metal ions to form functionalized MOFs. By adopting this approach, the catalytically active metal component is orderly embedded within the MOFs pores, effectively preventing the leakage and aggregation of the active catalytic substance during the catalytic process.

It has been demonstrated that loading metal nanoparticles (such as Pd, Ir, Ru, Co., among others) onto UiO-67 can substantially enhance the conversion of greenhouse gases like carbon dioxide and methane into non-hazardous gases. Additionally, numerous researchers have concentrated their efforts on transition metals like Au, Cu, Pd, Pt, and others, which exhibit exceptional performance in catalyzing reactions such as cyclopropanation, benzylic alcohol oxidation, and the Suzuki coupling/asymmetric aldol reactions. Furthermore, there are researchers who are intrigued by metal complexes such as Al, Ce, Ni, Fe, Mo, Ti, and others, which are loaded onto the surface of UiO-67 MOF and employed to catalyze reactions like the Morita-Baylis-Hillman reaction, Fenton-like reaction, as well as a range of photocatalytic and electrocatalytic reactions (Cao et al., 2020; Cheng et al., 2022; Zhuo et al., 2021; Li Y. et al., 2022; Lü et al., 2020; Masoomi et al., 2019). Based on our literature review, we observed that the majority of catalytic applications involving UiO-67 are predominantly in the photocatalytic domain, as detailed in Table 3. In a manner analogous to the preceding discussion, the current state of mechanistic research on UiO-67 series metal organic frameworks in photocatalytic reactions is somewhat lacking. Consequently, there is significant scope for in depth investigation into the internal electron and energy transfer processes within these materials during photocatalysis. This exploration can be conducted from multiple perspectives, including the transfer of electrons from organic ligands to the central metal, inter-atomic electron transfer within the central metal cluster, and the broader electron and energy transfer dynamics in the context of photocatalysis. Currently, researchers in the field of photocatalytic water splitting predominantly focus on the semi-reactive process of hydrogen production *via* water photolysis in the presence of a sacrificial agent. However, there is a notable dearth of studies investigating the complete decomposition of water using single MOFs. Beyond conventional strategies such as constructing heterojunctions or incorporating loaded catalysts, the challenge of achieving total photocatalytic water decomposition using a single MOF framework represents a pivotal research frontier. Overcoming this hurdle holds immense significance and research value, as it could unlock new possibilities for harnessing MOFs in water - splitting applications. These studies not only broaden the application scope of UiO-67 MOF materials in catalysis but also offer novel insights and solutions for addressing environmental challenges.

**TABLE 3:** Applications of UiO-67 series MOFs in photocatalytic field.

Materials	Synthesis method	Applications	Literatures
Eu^3+^-UiO-67	Solvothermal synthesis	Detect Ag^+^ Fluorescent Sensor	Luo et al. (2021)
Ru (bpy)_3_^2+^-UiO-67	Solvothermal synthesis	Immunosensor for the detection of hexestrol (DES)	(Dong et al., 2018)
Ru (bpy)_3_^2+^-UiO-67	Solvothermal synthesis	Therapeutic nanoplatform for *in vitro* two-photon fluorescence imaging and photodynamic therapy	Chen R. et al. (2017)
ZA-MPTMS-Eu-UiO-67	Solvothermal synthesis	Fluorescence detection of ammonia vapors	Ma and Yan (2019)
Ru(II)(bpy)_2_ (dcbpy)-doped UiO-67	Solvothermal synthesis	Ruthenium doping ratio and photocatalytic mechanism	Zhu et al. (2017), Maza et al. (2016), Maza et al. (2014)
Ru(dcbpy)-UiO-67	Solvothermal synthesis	Photocatalytic debromination of *α*-bromone	Santiago-Portillo et al. (2018)
UiO-67-ZnPc	Hydrothermal synthesis, Microwave method	Photocatalytic oxidation of naphthoquinones	Lü et al. (2020)
UiO-67-Ru	Hydrothermal synthesis	Photocatalytic synthesis of *β*-acetylaminopropenyl sulfone	Xu et al. (2020)
UiO-67	Solvothermal synthesis	Liquid Chromatography Fluorescence Detection	Liu et al. (2016)
SRB@UiO-66 SRB@UiO-67	Solvothermal synthesis	Good cellular dyes and laser materials	Ruan et al. (2020)
UiO-67	Solvothermal synthesis	Degradation of anionic organic dyes	Zhang et al. (2018)
UiO-67	Hydrothermal synthesis	photocatalytic hydrogenation	Sun et al. (2020)
CsPbX_3_@UiO-67	Solvothermal synthesis	White LED devices	Zhang et al. (2019)

## 5 Conclusion

We discovered that existing techniques possess the capability to precisely control the composition of nodes, the substitution pattern of connectors, and the defect density within modified UiO-67. Among the numerous known MOFs, UiO-67 distinguishes itself with exceptional properties: catalysts synthesized within its framework demonstrate remarkable selectivity and catalytic activity in various chemical reactions, capable of undergoing at least five rounds of recycling, thereby aligning perfectly with the principles of green chemistry (Hu et al., 2019; Valdebenito et al., 2022). However, we also acknowledge the limitations of the current study: (1) the majority of UiO-67 derivatives primarily catalyze relatively well-established reactions, with a notable lack of in-depth investigation into their catalytic mechanisms (Das et al., 2019; Zwolinski and Chmielewski, 2017). (2) during the catalyst recycling process, a certain amount of catalyst is inevitably lost due to operations such as extraction and filtration (Li et al., 2024). (3) The majority of MOFs have yet to be applied to industrial production on a large scale and are still in the nascent stages of commercialization. These are questions that need to be further explored in the next scientific studies. The potential of UiO-67 is, undeniably, geared towards fostering its broader utilization within the realm of multidisciplinary chemistry (Zhou et al., 2025). Presently, existing research endeavors are unevenly allocated across various applications, with a predominant emphasis on catalysis. Future research endeavors will concentrate on broadening the applications of the Zr-UiO-67 across diverse domains. This includes delving deeper into its utility in carbon dioxide capture and conversion, as well as organic transformations. Additionally, investigations will extend to its role in water treatment, environmental remediation, biosensing, serving as porous carriers, facilitating drug delivery, and enabling energy storage solutions. Extensive research centered around UiO-67 has demonstrated its boundless potential for commercial applications. Upon achieving practical implementation, this advancement will signify a monumental leap forward in the evolution of UiO-67 materials.
